# Acute kidney injury: a post-COVID-19 complication in children and adolescents

**DOI:** 10.1590/1984-0462/2025/43/2023171

**Published:** 2024-09-06

**Authors:** Maria Clara Mendes Maranhão, Marina do Nascimento Mateus, Giovanna Sturzenegger Tosatto, Érika Pangracio, Giovanna Zatelli Schreiner, Karen Previdi Olandoski, Renato Nisihara

**Affiliations:** a Universidade Positivo, Curitiba, PA, Brazil.

**Keywords:** COVID-19, SARS-CoV-2, Dialysis, Acute kidney injury, Pediatric nephrology, COVID-19, SARS-COV-2, Diálise, Lesão renal aguda, Nefrologia pediátrica

## Abstract

**Objective:**

To describe cases of acute kidney injury (AKI) in children diagnosed with COVID-19, associated risk factors, clinical aspects and outcome of cases.

**Methods:**

Retrospective study, carried out in a pediatric hospital between March 2020 and September 2021, with patients with COVID-19 who were diagnosed with AKI, studying information present in medical records such as comorbidities, age, gender and use of nephrotoxic medications.

**Results:**

We studied 40 cases, and male individuals were significantly more affected (62.5%; p=0.025). AKI was a severe complication of COVID-19 infection, with 100% of the sample requiring admission to the Intensive Care Unit and 22.5% dying. The most prevalent comorbidities analyzed in this study were epilepsy, cerebral palsy and heart disease. Most patients were classified according to Kidney Disease: Improving Global Outcomes (KDIGO) criteria as KDIGO 1 (42.5%), and required orotracheal intubation (67.5%). The frequency of use of nephrotoxic medications and need for dialysis was low, with percentages of 35 and 17.5%, respectively. Among the children who died, 70.4% had some comorbidity and 88.8% received invasive ventilation.

**Conclusions:**

AKI in children with COVID-19 infection is associated with severe conditions. Despite the severity, most patients were discharged alive from the hospital.

## RESUMO

**Objetivo:**

Descrever casos de lesão renal aguda (LRA) em crianças diagnosticadas com COVID-19, associando fatores de risco, aspectos clínicos e evolução dos casos.

**Métodos:**

Estudo retrospectivo, realizado em hospital pediátrico entre março de 2020 e setembro de 2021, com pacientes com COVID-19 diagnosticados com LRA, que examinou informações presentes em prontuários como comorbidades, idade, sexo e uso de medicações nefrotóxicas.

**Resultados:**

Foram estudados 40 casos, sendo o sexo masculino significativamente mais acometido (62,5%; p=0,025). A LRA foi uma complicação grave da infecção por COVID-19, com 100% da amostra necessitando de internação na Unidade de Terapia Intensiva e 22,5% indo a óbito. As comorbidades mais prevalentes analisadas neste estudo foram epilepsia, paralisia cerebral e cardiopatia. A maioria dos pacientes foi classificada pelos critérios *Kidney Disease: Improving Global Outcomes* (KDIGO) como KDIGO 1 (42,5%) e necessitou de intubação orotraqueal (67,5%). A frequência de uso de medicamentos nefrotóxicos e necessidade de diálise foi baixa, com percentuais de 35 e 17,5%, respectivamente. Entre as crianças que faleceram, 70,4% apresentavam alguma comorbidade e 88,8% receberam ventilação invasiva.

**Conclusões:**

A LRA em crianças com infecção por COVID-19 está associada a quadros graves, apesar de a maior parte dos pacientes ter recebido alta hospitalar.

## INTRODUCTION

Acute kidney injury (AKI) in children infected with SARS-CoV-2 has been described in younger children with overweight and comorbidities.^1^ According to Kari et al., who studied 89 children, 21% of pediatric patients admitted for hospital treatment of COVID-19 developed an AKI condition. Of these, 58% were diagnosed with stage I AKI, 31.5% with stage II AKI, and 10.5% with stage III.^1^ The diagnosis of AKI is made using the Kidney Disease: Improving Global Outcomes (KDIGO) criteria, the same as for patients not infected with COVID-19.^2^ However, in children, such a diagnosis is a challenge, as it depends on the values of serum creatinine and urinary output to define the stage of the injury, values that depend on the child’s age and muscle mass.^3^ Deep et al. reported that the main trigger for AKI in children is dehydration, and meticulous attention to the status of body fluids is essential.^4^

The impact of AKI on the outcome of those infected with COVID-19 is highlighted. Bjornstad et al. reported that mortality in patients infected with COVID-19 was 3.4 times higher in those who developed AKI.^5,6^ Thus, this study aimed to identify the demographic profile (gender, age and comorbidities), the clinical and hospitalization complications (need for dialysis and invasive mechanical ventilation) of pediatric patients who developed AKI after being diagnosed with COVID-19, the risk factors for the occurrence of AKI and the clinical outcomes.

## METHOD

The study was approved by the Research Ethics Committee of the institution under protocol number 5.005.944 and Certificate of Presentation for Ethical Appreciation (CAAE) 51771921.8.0000.0097, in respect of secrecy and confidentiality rules, guaranteeing the anonymity of the researched individuals.

This is a descriptive retrospective study of a series of cases, with patients admitted to a tertiary reference pediatric hospital in Curitiba, Paraná, Brazil, from March 2020 to September 2021. Were included patients aged less than 18 years who developed AKI after a diagnosis of SARS-CoV-2 infection confirmed by reverse transcription polymerase chain reaction (RT-PCR) and were under the care of the pediatric nephrology service. Those patients who had incomplete electronic medical records and those with previous chronic renal failure were excluded from the study.

The electronic medical records of patients with N17 (Acute Renal Failure) according to the 10^th^ Revision of the International Classification of Diseases (ICD-10) were analyzed. The review of medical records collected data on sex, age at diagnosis, comorbidities, date of diagnosis of COVID-19, days of hospitalization in total and in the Intensive Care Unit (ICU), signs and symptoms related to AKI, complications, use of nephrotoxic drugs, KDIGO, need or not of dialysis therapy, dialysis days and outcomes of the patients involved in the study. Outcomes were classified as discharge or death.

For analysis purposes, patients were divided into the following age groups: from zero to one year, from two to four years, from five to ten years and from 11 to 18 years.

The KDIGO classification^2^ was used to evaluate the prognostic and severity of AKI, diagnosed when there was an increase in serum creatinine (SCr) of ≥0.3 mg/dL within 48 hours or ≥50% within 7 days OR a urine output of <0.5 mL/kg/hour for >6 hours.^2^

KDIGO scores were calculated and classified as stage 1 (mild); 2 (moderate); and 3 (severe) renal injury.^2^

- Stage 1: ↑ SCr ≥ 0.3 mg/dL or ↑ SCr ≥1.5- to 2.0-fold from baseline;
- Stage 2: ↑ SCr >2.0- to 3.0-fold from baseline;
- Stage 3: ↑ SCr >3.0-fold from baseline or SCr ≥4.0 mg/dL with an acute ↑ of ≥0.3 mg/dL or initiation of renal replacement therapy.

Data was displayed in frequency and contingency tables. Frequency was expressed in percentages. Data was distributed using the Shapiro-Wilk test and central tendency was expressed in mean and standard deviation — SD (parametric data). Nominal data was compared using the Fisher and chi-square tests, while numerical data were compared using the unpaired *t*-test or Mann-Whitney test. P-values lower than 5% were considered statistically significant. The tests were performed using the software GraphPad Prism version 6.0.0 for Windows, GraphPad Software, San Diego, California USA.

## RESULTS

During the study period, 40 cases of AKI were found in pediatric patients with COVID-19 who met the criteria used in our study. Two patients who had incomplete electronic medical records and one with previous chronic renal failure were excluded from the study.

The demographic and clinical data of the patients are available on Table 1. Males were significantly more affected (62.5%; p=0.025) and, regarding origin, 70% of the cases lived in the capital and 30% were from the interior of the state. As for the distribution of ages, 30% of the study population were under two years of age, 12.5% were between two and four, 22.5% between five and ten and 35% over 11 and up to 18.

**Table 1:** Demographic and clinical data of studied patients (n=40).

Variable	N	%
Sex		
Female	15	37.5
Male	25	62.5
Age groups (years)		
0–1	12	30
2–4	5	12.5
5–10	9	22.5
11–18	14	35
City of residence		
Capital	16	40
Metropolitan area	12	30
Countryside	12	30
Comorbidities		
Epilepsy	8	20
Heart diseases	5	12.5
Cerebral palsy	5	12.5
Asthma	3	7.5
Down syndrome	3	7.5
Immunodeficiencies	3	7.5
Microcephaly	2	5
Myelomeningocele	2	5
Oncological diseases	2	5
Liver disease	1	2.5
Diabetes Mellitus type 1	1	2.5
Without comorbidities	10	25
Outcome		
Discharge	31	77.5
Death	9	22.5
Need for intubation		
Yes	27	67.5
KDIGO		
1	17	42.5
2	11	27.5
3	12	30
Need for dialysis		
Yes	7	17.5
Exposure to nephrotoxic drugs		
Yes	14	35

KDIGO: Kidney Disease: Improving Global Outcomes.

Clinical and hospitalization data about the studied population are available in Table 1, with a median hospital stay of 23 days (interquartile range — IQR=14–41 days). All patients, at some moment during hospitalization, went to the pediatric ICU, with a median period of 11 days (IQR=7–18 days) in these beds. Most patients were discharged from the hospital (77.5%), had a KDIGO stage one (42.5%) and did not require dialysis (82.5%). No use of nephrotoxic medication was observed in 65% of the sample. There was a need for orotracheal intubation (OTI) in 67.5% of the cases. After one year of the analyzed period, 15 children (37.5%) returned to the service in the outpatient or emergency modality, but for other reasons not associated with previous AKI.

The KDIGO classification showed stage 1 in 19/40 (47.5%) of cases, stage 2 in 9/40 (22.5%) and stage 3 in 12/40 (30%) of the children. Seven patients in stage 3 need dialysis, with a median of dialysis equal to 15 days. Among the patients in stage 3, eight (66.7%) died.

Regarding the nine deaths of the studied population (22.5%), the majority were male (66.6%), with a KDIGO stage 3 (88.8%), required dialysis (66.6%) and needed OTI (88.8%). One third of patients who died had some heart disease. Only one male who died had no comorbidity. He was a 17-year-old with sepsis, used polymyxin B, had 13 days of hemodialysis and 25 total hospitalization days, all in the ICU. The extremes of age were also the most affected, as displayed in Table 2.

**Table 2:** Data of patients who died (n=9).

Variable	N	%
Sex		
Female	3	33.3
Male	6	66.6
Age groups (years)		
0–1	4	44.4
2–10	2	22.2
11–18	3	33.3
Comorbidities		
Epilepsy	2	22.2
Down syndrome	2	22.2
Other^*^	3	33.3
Without comorbidities	1	11.1
Cause of death		
Cardiorespiratory arrest	6	66.6
Brain death	1	11.1
Central Nervous System bleeding	1	11.1
Multiple organ failure	1	11.1
Exposure to nephrotoxic drugs		
Yes	4	44.5
KDIGO		
1	1	11.2
2	0	0
3	8	88.8
Need for dialysis		
Yes	6	66.6
Need for intubation		
Yes	8	88.8

KDIGO: Kidney Disease: Improving Global Outcomes.

*Immunodeficiency, cancer and cerebral palsy — one case each.

## DISCUSSION

This study presents a series of Brazilian pediatric cases that had COVID-19 and AKI, showing the severity of the concomitant diseases in these patients. Tripath et al.,^7^ in a meta-analysis, reported that the proportion of pediatric patients with multisystem inflammatory syndrome due to COVID-19 who developed AKI was 20% (95%CI 14–28%), and this finding is associated with greater chances of death.

Although the pathophysiology of AKI in patients with COVID-19 is still uncertain, it is believed that the mechanisms that contribute to the evolution of this condition are both renal and pre-renal, such as dehydration, cytokine storm, direct viral injury to the proximal tubules and use of nephrotoxic drugs.^4^ Dehydration results from vomiting, diarrhea, decreased fluid intake and administration of diuretics.^8^ The cytokine storm syndrome is mainly due to the increase in interleukin-6, with a cellular inflammatory response, and evolves with renal damage resulting from inflammation of the parenchyma.^9^ In addition to extreme inflammation, several systems are affected, jointly producing a combination of factors that lead to AKI.^9^ Furthermore, the greater expression of ACE2 receptors in the kidneys may be involved in the infection and this organ is directly affected by the virus.^10,11^

In our study, most cases were male, and children as a group were more affected, especially infants in the first year of life. In addition, around 1/3 of sample were adolescents between 11 and 18 years old, which is in line with the literature.^12,13^ Raina et al., studying 2,546 American children admitted to the ICU due to COVID-19, observed that 10.8% had AKI. Among these, 51.8% were male and 55.5% were between 12 and 18 years old.^12^ The highest prevalence of males may be related to the greater activity of ACE2 receptors in men, who, according to some authors, have a greater number of infections as well as greater severity of symptoms and mortality.^13^ In addition, it has been demonstrated that there is a 1.9 times greater receptor activity in the kidneys of individuals of this genre, as verified in an experimental study.^14^

Regarding comorbidities, in the studied sample, 12.5% had underlying heart disease; less than the 58.8% reported in a study carried out in the USA.^12^ It is noteworthy that, among the three patients with Down syndrome hospitalized due to AKI, two died.

Concerning the KDIGO classification for AKI, we found that 42.5% patients were classified as KDIGO stage 1 and 30% were classified as stage 3. Kari et al. found that, among children with AKI during hospitalization due to COVID-19, 58% were in stage 1, 31.5% stage 2 and 10.5% in stage 3.^1^ These data indicate that the patients in our sample had more severe AKI than those found in the American study.^1^ Genetic and environmental factors may be involved in this finding.

The use of nephrotoxic medications was very common in the treatment of COVID-19 and may be associated with a higher risk of developing AKI.^9^ Among the 40 patients analyzed, about a third were using nephrotoxic medications during hospitalization. Chopra et al. reported that 66.7% of the studied sample were using these medications.^15^ The sample size of the present study did not make it possible to associate the use of a specific medication with a higher probability of AKI.

This study had some limitations, such as its retrospective design, the analysis of data from a single hospital center and the small sample size, and did not allow other statistical analyses such as risk factors for AKI and death. However, it was possible to show with regard to COVID-19 in pediatric patients that, despite there being a significantly lower number of hospitalizations when compared to the adult population, outcomes are potentially severe.

In conclusion, AKI in children and adolescents with COVID-19 is related to severe conditions and the need for ICU admission, despite most of the sample having been discharged from the hospital.

## Data Availability

The database that originated the article is available with the corresponding author.
